# The Hospital Nursing Supplement

**Published:** 1896-02-08

**Authors:** 


### "The Hospital" Nursing Mirror.

Being the Extra Nursing Supplement of "The Hospital" Newspaper.

[Contributions for this Supplement should be addressed to the Editor, The Hospital, 428, Strand, London, W.C., and should have the word "Nursing" plainly written in left-hand top corner of the envelope.]

### News from the Nursing World.

### THE PATIENTS' FRIENDS.

It was a kind thought of the authorities at St. Thomas's Hospital to allow the wards to be illuminated during visitors' hour on Christmas Day, and the charming sight will not easily be forgotten by the patients' friends. Another pleasant concession was deeply appreciated by the patients, who, to their great delight, had each the privilege of asking one friend to remain to tea with them. After this daintily-served meal was over various concerts took place, and a truly happy Christmas was passed by the inmates of a hospital whose fine proportions admit of entertainments impossible in institutions of less ample dimensions.

### HAMPSTEAD NURSING ASSOCIATION.

The Hampstead Nursing Association, established to supply skilled nurses to the sick poor in their own homes, is at present sorely in need of pecuniary assistance. Each year the demands for the services of the nurses show a marked increase, and the work is growing more rapidly than the subscription list, which ought at least to keep pace with it. The nurses carry on their duties under the immediate direction of medical men, and are all said to be fully trained before they take up this valuable branch of nursing. The record of the cases which have been visited and cared for already is a long one, and great would be the lamentations of the sick poor of Hampstead if the present year's financial position necessitated the curtailment, in any direction, of the society's useful work.

### MISLEAD NG ADVERTISEMENTS.

Our attention is frequently called to a certain class of advertisement framed, apparently, to deceive and disappoint hospital sisters, assistant matrons, and charge nurses. It takes the form of an announcement that some post of matron or superintendent of nurses will be shortly vacant, and it invites applications from highly-trained ladies of experience. If the offered salary be fairly good and the locality and reputation of the institution satisfactory, scores of women are encouraged to apply. To do this they must first get testimonials from the matron, the committee, and medical staff of the establishment they are in, and this procedure, of course, necessitates an explanation of their intention to leave. After all the formalities have been complied with, a number of candidates are asked to come before the Selection Committee. It is then not infrequently announced that choice has been already made of a local candidate or a relation of one of the officials. This person may be the best for the post, or she may not, but she has probably canvassed every governor before the first insertion of the advertisement. The injustice of the whole procedure will long rankle in the minds of ladies who have been invited to go through the farce of applying for the post which none of them were intended to get. An election in which all candidates have equal chances is admirable, but when appointments are forgone conclusions it is a sorry joke to advertise them as vacant and cause trouble, expense, and disappointment to many eligible candidates.

### A LOSS TO MILL ROAD INFIRMARY.

It is with regret that we learn that Miss Edith Walker has resigned the post of matron at Mill Road Infirmary, Liverpool, for we know that the difficulty of securing fully-trained and experienced nurses to take work under the Poor Law is a great obstacle to the development of proper training schools in infirmaries. Miss Walker has been exceptionally successful as an organiser, and the arrangements which she has carried out in the nursing and domestic departments at Mill Road have earned for her and for the training school inaugurated under her auspices no inconsiderable credit. On many sides we have heard unstinted praise of the admirable order of the beautiful new infirmary and of the good tone of the probationers in training there. Although it cannot but be difficult to find an equally competent successor, yet the present matron has the satisfaction of knowing that no future officer will encounter the discomforts which accompanied her energetic introduction of a modern system of nursing. When Miss Walker leaves the infirmary she will carry with her good wishes from a large circle of outside friends, as well as from a loyal band of nurses by whom she will be sorely missed. The first batch of probationers passed their examination well some three weeks ago, and their certificates were signed by the medical superintendent, Dr. Chapman; Dr. Newbold, the examiner; Miss Walker (matron), and the Clerk to the Guardians. The introduction of an examiner unconnected with the infirmary is an excellent plan and worthy of imitation.

### DYING AND DANCING.

The reports of nurses' dances in workhouse infirmaries which still crop up in the press as a kind of after-glow of the Christmas festivities show that some of these Poor Law establishments are still blind to the social status of modern nurses. Probably in former days a ball in which the officers of both sexes took part harmonised with existing conditions. Happily things are changed now, and the nurses attached to any decent training school would shrink from the thought of such a ball heing given nominally for their pleasure. Judging from the comments circulated about these infirmary dances, it seems probable that junior officers in the employ of the Poor Law find these parties (ostensibly given for the nurses) most convenient opportunities for entertaining their own friends. There can be no two opinions as to the undesirability of such entertainments being sanctioned by medical superintendents who are answerable to the Local Government Board for the discipline of infirmaries. No self-respecting nurse desires a lower standard than that which has now been attained in the best hospital training schools. There are many ways of giving nurses pleasure outside the institutions in which they work, and these could be easily learnt if Guardians consulted the ladies in charge of their nurses instead of accepting the code of ethics laid down by inferior and interested officers. So long as the Local Government Board fails to recognise the futility of appointing superintendent nurses to whom no power is given to protect their probationers, work in Poor Law infirmaries must be disheartening to those educated women who are from time to time brave enough to face its difficulties.

### MORE WORK WANTED.

In contrast to the overworked condition of some district nurses, the Portsmouth Association for Nursing the Sick Poor seems to have too little to do. This is apparently not the result of any absence of illness in the district, but rather of local ignorance respecting the resources of the association, which is a flourishing one.

### DARLINGTON DISTRICT NURSES.

The Darlington District Nursing Association has done a year's hard work well, and it was shown at the annual meeting that still further financial support is needed. Four Queen's nurses would find plenty to do, and the funds are inadequate for this number; but it is unlikely that subscriptions will fail now that the usefulness of the association has been so clearly demonstrated. The speakers at the meeting, presided over by the Mayor, testified to the popularity of these Queen's nurses with the patients, and also to the doctors' appreciation of their services.

### THE LATE MISS STEWART.

Miss Stewart, of the Blythswood Training Home, Glasgow, whose death on the 14th Jan. was lamented by a large circle of friends, was for twenty years well known to the nursing world. Miss Stewart joined Miss MacAlpin at the Glasgow Training Home for Nurses in 1875, and for fourteen years was fully occupied with the nursing of the private patients and the training of probationers. In 1889 Miss Stewart left the home and took a house herself, where her staff of nurses was gradually added to until she had some thirty women working under her.

### RAINTON DISTRICT NURSE.

The absence—on account of illness—of Lady Londonderry from the annual meeting of the Rainton District Nursing Association was a cause of great regret to those who knew the deep interest which she has from the first taken in the movement. Pleasant rooms are provided for the nurses' accommodation by Lady Londonderry, and the secretary referred to her kind generosity in the report laid before the meeting. Great appreciation of Nurse Rudd's services was exhibited by various speakers, who cordially acknowledged her special fitness for the post of district nurse.

### DISTRICT NURSING AT MONTROSE.

The Montrose Nursing Association, affiliated with the Queen's Jubilee Institute, has submitted a satisfactory annual report to the subscribers. Nurse Armstrong paid 2,774 visits during the year, and her services have been greatly appreciated by her patients. Amongst other useful gifts received by the association a large number of articles of clothing were provided by the Forfarshire branch of the Scottish Needlework Guild.

### PROOF WANTED.

It would be interesting to know the source from which an anonymous correspondent derived the information which he has contributed to the *Glasgow Herald* under the title "Nurses' Grievances." Unless the writer is prepared to give the name of the nstitution, or to furnish some proofs of the accuracy of his assertions, nurses in general will be inclined to believe that some humorist has diverted him or herself at the expense of this would-be champion. Amongst other accusations, it is stated that in some instances the nurses' meals are served in the same room where operations are performed; single beds have to hold two nurses; nurses on night duty have to occupy the beds which have been slept in by the day nurses; servants have to sleep in the rooms where cancer dressings are burnt and bandages are hung up to dry night and day. If these horrible practices exist in any institution in Scotland or England, the Glasgow correspondent should certainly quote the establishment by name. It is neither fair nor wise to write as if such experiences were the lot of nurses elsewhere.

### NURSING IN BLAIRGOWRIE AND RATTRAY.

The Blairgowrie and Rattray District Nursing Association placed an admirable report before the annual meeting of subscribers. The work of Nurse Fleming is appreciated by patients and doctor, and her duties have extended so rapidly that a nurse has been temporarily engaged to assist through the winter months. When the funds permit of a permanent addition to the staff it will, no doubt, be gladly made by the committee, who take much interest in the success of the association.

### SHORT ITEMS.

A trained nurse has been engaged by the Earl and Countess of Warwick to attend cases of illness in the colliers' families at High Littleton, Farnborough, and Chilton.—*Nursing Notes* for February contains some interesting articles, and the correspondence columns under the heading "Nurses in Council" contain, as usual, many valuable hints.—A representative committee has been appointed at Castleford to consider schemes for a cottage hospital or for securing district nurses.—The annual meeting of the Radstock Sick Nursing Society was not well attended, but the appeal for better financial support deserves local consideration, the work of the nurse being needed and valued.—The Shoreditch Guardians have shown their appreciation of the Haggerston and Hoxton District Nursing Association by voting that their annual subscription should be increased to fifteen guineas.

### Lectures on Nursing.

By a Superintendent of Nurses.

### IX.—FEEDING.

It seems to me an essential part of a nurse's duties to learn how important it is to give food wisely and in a tempting form. Soon after I became a nurse I was greatly gratified by a speech made by a boy who was very ill, and not easily pleased. I had been delayed by hospital business one morning, and came to my ward a few minutes later than usual. On going near this patient's bed, he said to me, in an irritable tone, "Oh, nurse, how late you are! I have been waiting ever so long for my breakfast. You know I won't let anybody else give it to me; no one cuts bread and butter like you do." Though I reproved him for his impatience, it pleased me very much to receive this word of commendation.

I have often been amazed at the thoughtlessness nurses display in the giving of meals to the sick. I have seen great slices of thick bread and butter handed to a person who would perhaps take just one bite of a slice and leave all the rest, and I have thought how different it would have been, if the nurse had given him two or three dainty little slices instead; these, in all probability, would have been eaten with relish. Then, again, I have seen toast made—such toast! Clumsy pieces of bread with the crust left on, and perhaps burnt or dried till there was very little taste of bread remaining. Sometimes I have noticed a patient, with a "finicky" appetite, staring at a plate piled up with large, thick slices of meat and a heap of greens and potatoes, and I have said to myself, "If that plate had been put before me I should have felt sickened at the sight of it, before making an attempt to eat the food." Another time I have watched a nurse hand a basin of beef tea or broth to someone on low diet, never observing that it was swimming with grease. If you really love your work, do consider these details, which appear so small and yet are so important to invalids. See that the crockery, knives, forks, and spoons you take to your patient are clean, that the tray cloth is unsoiled, that he has salt and all that he requires without having to wait for it. Take care that food intended to be hot is hot; make everything look as appetising as possible.

Never say to a sick man, "Don't you think you could take this or that?" unless you have it at hand all ready for him. Very often some fresh article of diet brought suddenly to a patient with a quiet, cheery "Now, then! I have something for you I think you will like," will result in his taking it at once and enjoying it, whereas if it has been talked about, and the patient has for the moment fancied it, by the time it has been cooked all desire for it will have passed away. Do not hurry a helpless patient through his meals, and always put your hand under his pillow and raise his head whilst he is eating.

Do not be afraid of letting patients drink water. Anyone who has not been ill can hardly realise how deliciously refreshing cold water is to a sick man. It should, however, be given in small quantities at a time, and the nurse should make quite sure of its being perfectly fresh, and should never take it from a bottle which has been standing long in the room. If it is fresh let him have it as often as he likes, unless contrary orders have been given by the medical man. Semi-conscious people are often very thirsty, so they should have water or liquid of some kind pretty frequently.

A nurse should always try to find out the peculiar likes and dislikes of her patient as to food. There is an old saying, "What is one man's food is another man's poison," and surely it is very true. Mutton is supposed to be the most easily-digested meat, and yet there are some people who dare not touch it. In G. H. Lewis's "Physiology of Common Life," he mentions the case of the Abbé de Villedieu, to whom mutton proved a poisonous food. From his earliest years he had an intense repugnance to it, and "neither the entreaties of his parents nor the menaces of his tutors could induce him to overcome it. After reaching the age of thirty on a regimen of vegetable food, he was over-persuaded, and tried the effect of meat soups, which led to his eating both mutton and beef; but the change was fatal, plethora and sleepiness supervened, and he died of cerebral inflammation.' The same book tells us that Dr. Prout knew a person on whom mutton acted as a poison. "He could not eat mutton in any form. The peculiarity was supposed to be owing to caprice, but the mutton was repeatedly disguised and given to him unknown, but uniformly with the same result of producing violent vomiting and diarrhœa. And from the severity of the effects, which were, in fact, those of a virulent poison, there can be little doubt that if the use of mutton had been persisted in, it would soon have destroyed the life of the individual."

These facts should make us very careful not to urge upon children food they may have an intense repugnance to, such as fat, eggs, milk, vegetables, or fruit. I once knew a man who so disliked the smell of vegetables that he would cross over the road to avoid passing a greengrocer's shop, and we have all of us, no doubt, known persons who have had to us an unaccountable antipathy to some or other article of diet. I think doctors sometimes err by not considering the idiosyncrasies of their patients, and nurses should certainly do their best to find out any peculiarities of this kind they may have, and acquaint the medical man in charge.

A few years ago a lady with whom I am familiar was asked to go down to the country to see a friend who was very seriously ill, and her life almost despaired of. It was said she would not do as the doctor wished her, and it was hoped that a visit from one accustomed to hospital life might be of use. The lady I refer to started off as soon as she could, and found her friend so ill that she felt there was little hope for her. She learned that the doctor wished the patient to take milk, with egg and brandy beaten up with it, and bread and milk in addition, but with the result of causing sickness after every meal, and constant diarrhœa. The poor sick woman, always thin, scarcely seemed to have any flesh left on her bones, and was a pitiable sight. Knowing that from a girl, eggs, however disguised, always disagreed with her, that milk was too heavy for her, and that brandy always sickened her, my friend at once set to work to think what could be done in the way of altering her diet. She drew up a little diet card, and arranged that the lightest food possible, and in small quantities, should be given constantly, and at regular and short intervals; and to her joy the diarrhœa abated, the sickness stopped, and in a very short time she had the satisfaction of hearing the doctor say to his patient, "Well, I really think I can say you are better." He was very good, and left her feeding henceforth in the hands of the patient's friends, who felt they had had a great deal to do with "pulling her through." When her recovery was assured, one of the things she found very useful was Revalenta Arabica, made with beef tea. This most useful flour was in great demand some years ago, but, like so many good things, has had its day and seems almost forgotten, and yet it certainly is an excellent food, and can be cooked in a variety of ways. I know a lady who suffered terribly from dyspepsia, and for more than a year lived almost entirely upon it, enjoying during that time fairly good health and freedom from pain. It is made from lentils, but baked and ground, and prepared in such a way that it is most easily digested. It can be made with milk, beef tea, veal or chicken broth. The last is the lightest and most delicate way of preparing it, and great care should be taken that all grease be skimmed off the top. It is better not to take a large quantity at a time, even if the patient can eat it. A breakfast-cup full is sufficient for one meal, and this remark applies, I think, to everything given to a patient. If the quantity is the least bit more than he fancies he will probably take a dislike to the nicest thing imaginable, and turn from it in future with disgust.

Few people appreciate the value of coffee, and fewer still know how to make it. If strong it acts as a splendid stimulant, and the aroma is most appetising. For one person a large tablespoonful of freshly-ground coffee should be used; three ounces of boiling water being poured on. It should be made in a percolator if one is obtainable, if not, it can be strained through an ordinary penny strainer. Pour into a cup three parts filled with hot milk. Thus you have a most nourishing refreshing drink.

### The Nurses' Co-operation.

Dr. H. P. Hawkins, treasurer to the Co-operation, writes: On behalf of the Nurses' Co-operation, I wish to express our thanks for the kindly article concerning our work which appeared in your last issue. It needs, however, correction on one point. The deduction from the nurses' earnings still remains at the rate of 7½ per cent. The question of reducing this was fully considered at a meeting of the nurses some two years ago. The experience which had been gained up to that time taught us that the ordinary expenses of management could be met, possibly, by a deduction of 6 per cent., and certainly by a deduction of 6½ per cent. It was thought, however, to be safer to maintain the rate at 7½ per cent., so that some reserve fund might be accumulated with which to meet any extraordinary expenditure that might arise. The wisdom of this decision of the nurses has, as a matter of fact, already been proved. I may say, in addition, that the expense of management does not increase at the same rate as the income, and it is therefore conceivable that a lower rate of deduction may eventually be found possible and safe, if the Nurses' Co-operation should grow into the extensive organisation which its founders hoped for, and which your article foreshadows.

[We have much pleasure in publishing Dr. Hawkins' letter. A deduction of 1s. 6d. in the pound is not a large amount, and must be willingly submitted to by the nurses in the Co-operation, seeing that it enables them to accumulate a considerable reserve fund, which will place them in a position to do great things for themselves and for nursing in the near future. We hope that every nurse now living will study the history of the Nurses' Co-operation, that she may realise the difference between institutions established with the sole object of helping nurses to help themselves, and of institutions which owe their origin to a desire on the part of some individual or individuals to obtain the command of money, to make a position for themselves, or to put money into the promoters' pockets at the expense of the nurses who may become associated with the latter type of institution. We have little sympathy with those who declare that they were deceived because they did not understand. Nurses ought to make it their business to understand. The paths of knowledge are open to every nurse nowadays, and every nurse ought to enter and pursue them until she is able to guard herself from deception and consequent loss. We have received a letter from another correspondent calling our attention to the statement in question, to which the foregoing will reply.—Ed. *T. H.*]

### Lady Cyclists "on a Firm Social Basis."

"To cycle or not to cycle?" that is the question of the hour for ladies of all ranks and ages. Medicine has been appealed to, and, as is not unusual, "doctors have differed." Where the luminaries of medical science have almost "feared to tread," fashion, that great arbiter of feminine destiny, has rushed in and settled the matter out of hand. We learn from a contemporary that at Florence the value of cycling, as a "therapeutic agent," has been for some time recognised by the British physicians who practise in the sunny South, and that, probably as a consequence, a few ladies, with the Countess de Talleyrand at their head, have cycled bravely in spite of Mrs. Grundy's nods and frowns and most solemn disapprobation, All that is now changed, however. This season no less a person than the British Consul-General, accompanied by his daughter, has lent the weight of his example to the charming pastime, with the result that cycling for ladies is now at last established "on a firm social basis." This is distinctly good. For ourselves, we have always made little of the social aspect of the question, and insisted that for women in good health, and of a suitable age, it is difficult to imagine a more invigorating or pleasure-giving amusement than that of cycling. There is now a prospect of finally eliminating the "new woman." Women who cycle will be vigorous in body and wholesome in mind. Their native common sense, made robust by an active outdoor life, will clear their minds of the cant which is so fascinating to weak novelists and "penny cyclopædia" scientists. The woman on wheels will prove herself much more than a match for the unclean female cultivator of nasty moral microbes.

### Our American Letter.

### News from New York.

Another hospital, the Fordham Hospital, has been lately added to the list of those institutions nursed by the New York Training School for Nurses, Blackwell's Island. This makes the fifth, those already attached being the City and Maternity Hospitals, Blackwell's Island, and the Harlem and Gouverneur Hospitals, New York City. A two years' course of training is offered to candidates, during which time they live at the pleasant home on Blackwell's Island, serving in the wards of the various hospitals connected with the school as required. Another new feature in connection with this training school is the recent establishment of an Advisory Board, its members being several well-known residents in New York. This departure is welcomed as a valuable token of interest in a municipal hospital on the part of the general public.

Mrs. Quintard, recently superintendent of the Connecticut Training School for Nurses, New Haven, began her new work on January 1st of this year, as superintendent at St. Luke's Training School in New York.

A piece of news of a more domestic character will no doubt be interesting to those to whom, before her marriage, the name of Miss Isabel Hampton was familiar, as the superintendent of nurses at the Johns Hopkins Hospital, Baltimore. A little son was born to Dr. and Mrs. J. Hunter Robb on Christmas Day, by all accounts a fine specimen of babyhood.

### The Royal British Nurses' Association and its Difficulties.

BY SIR DYCE DUCKWORTH, M.D.

WE have received the following communication from Sir Dyce Duckworth, and have pleasure in publishing it at his request:—

THE DOCTORS' VIEW.

It is an open secret that the affairs of the Royal British Nursing Association are carried on under peculiar difficulties, and it is also certain that the nurse-members generally are little aware of the nature of them. In this letter I ask your permission to make public some facts which have come within my own knowledge while I have been a member of the executive committee of that Association during the last three or four years.

The essential difficulty which has arrested the progress of much good work on behalf of nurses has been the presence in the governing body of a small clique of persons who have striven by all means in their power to wrest the management of the Association from the general body, and to place it in their own hands. The chief object of this persistent movement has been to lessen, and nullify if possible, the direction of the medical men in its councils, and so to emancipate the vocation of nursing from their control. Although such a condition is impossible, and can never be tolerated for a moment, efforts have, nevertheless, been made by this small clique to punish the executive for doing its duty, and to render the work of the Association nugatory. The officials have been hampered, maligned, insulted, and their least and most trifling errors in matters of small detail magnified and misrepresented as breaches of principle with a persistency, captiousness, and unkindness that would hardly be credited by rational people. This small clique considers itself to be composed alone of persons who are the friends of nurses or the "leaders of the nursing profession," and who declare that they, and only they, are the guardians of the "rights" and interests of nurses. Those who differ from them are styled "traitors," "dishonest," and "autocrats." These "leaders" show their tender regard for these so-called "rights" and interests by vilifying and misrepresenting the conduct of their colleagues in the nursing press, and no less by insulting the officers of the Association in the same channels. When outvoted, they refuse to take part in the work and duties they have been elected to carry out. Some of them stayed away from the Executive Committee for six months, and left their colleagues, chiefly busy medical men, to do their duty for them, subsequently taunting them, and pleading ignorance of the measures decided upon during their voluntary absence, charging them with trampling on the rights and interests of nurses which these particular representatives were bound to attend to, and to safeguard by their diligent attendance. But in truth the Executive Committee is quite innocent of having neglected its duties, or of having interfered in any way with the legitimate rights of any member of the Association.

Some members of this clique have withdrawn or curtailed their promised subscriptions to the funds in order to accentuate their displeasure. Having several times threatened to take legal proceedings against their colleagues singly or collectively, some of them lately took advantage of an unhappily expressed letter from the hon. medical secretary to a nurse who had been but a few hours a member of the Association, and who was so foolish as to write an improper and most misleading letter to a nurses' journal reflecting on the conduct of the office business, to drag the Association into the law courts. They secured their costs only on a technical legal point—the merits of the case having been subjected to no judicial decision—and thus caused a loss to the Association of £150 of the nurses' money.

These are some of the acts of these self-styled "leaders," who appeal by these feline amenities to the general body of nurses to support and follow them. They think to wear out the patience and good nature of the governing body, but they are grievously mistaken. The overwhelming vote of last Tuesday testified abundantly to the discredit that has come upon them, a vote that was not, and could not be, challenged. Nurse-members of the Association have only to be made fully aware of the tactics that have been carried on in their name to rally loyally on the side of those who can have no other object than to further their true interests and welfare. Those interests entirely depend upon the direction of nursing, as an occupation, by medical men and experienced nurses of administrative capacity. There are no personal interests to be served in such a work as this; nor are the public interests to be lost sight of. The governing body of the Association, acting under its charter, has to pay due regard to what is best for the medical profession in carrying on its daily duties, for the nurses as a body, and for the public, whose servants they are in this matter. The exalted ideals now prevalent for nurses are many of them out of place, and are leading to no little discontent both in the minds of the medical profession and of the public. Nurses have been rather too much "in evidence" of late; they have been petted and pampered, and some of them are disposed to turn and rend those who have spent time, thought, and money in helping them to the positions they now occupy. Many of these strange and impracticable ideas are, I believe, launched in a body known as the Matrons' Council, a body uncertified and undirected by medical men, and whose views, therefore, demand very searching criticism by the latter before they are submitted for general adoption.

THE NURSES' VIEW.

"A SUPERINTENDENT OF NURSES" writes: In another journal of last week I see a member of the Royal British Nurses' Association writing about the meeting on January 28th. I am very sorry that such a meeting should have been necessary, and still more so for the discordant tone of a small minority. I feel with our Royal President that unless loyalty first prevails, there never will be peace or honour. The member, in writing to the paper, absolutely forgot that even in her letter she was sowing discord and disloyalty in the Association. Still, I have faith to believe that the loyal nurses and their authorities will unite more than ever, so that the Association will not be allowed to suffer in the end.

### A CORRECTION.

DR. HEYWOOD SMITH writes: I see with astonishment in your issue of to-day, in the supplement, page cliii., that I am credited with a speech I never made. The mistake doubtless arose from the fact that Dr. Outterson Wood, who made that speech, was standing close to me at the meeting. What I did say was that I seconded Dr. Norman Kerr's amendment as it excluded the personal element from the original resolution.

### Where to Go.

TRAINED NURSES' CLUB.—Lecture, at a quarter to eight on February 28th, by Dr. Leith Napier; subject, "Mental Disturbances in the Pregnant and Parturient Woman."

ROYAL BRITISH NURSES' ASSOCIATION, 17, Old Cavendish Street, W.—Thursday, February 13th, at three p.m., Dr. Colman will lecture on "Electrical Treatment."

ROYAL INSTITUTE OF PAINTERS IN WATER-COLOURS, Piccadilly.—Ball in aid of the building fund of the Royal Ear Hospital, Soho, on February 12th. Duchess of Teck, patron. Particulars to be obtained from secretary, at the hospital.

ROYAL BRITISH NURSES' ASSOCIATION.—The course of lectures, by Dr. Colman, on the "Nursing of Nervous Diseases," will be continued on Thursday, February 13th, at three p.m., at 17, Old Cavendish Street, W. Dr. Colman has generously offered to give an additional lecture on Thursday, February 20th, at three p.m., on which occasion members and visitors alike will be admitted free.

### Workhouse Infirmary and Hospital Visiting.

By a Lady Visitor of Ten Years' Experience.

During all the earlier years of my life I had longed to be a visitor at a workhouse or hospital, and felt what vast experience I should gain thereby, and how useful I might make my experience to other people; but now that I have for some ten years past been a visitor both at the hospital and the workhouse women's infirmary of one of our largest towns, I feel I have sadly wasted my opportunities, and gone about the duties of a visitor in a lamentably amateurish kind of way. My only excuse for this is that I began as an amateur. I never was informed when I ceased to be one; and so I never knew when I ought to begin to brace myself up with strong bands of official red tape, and to conform to rules which I was supposed to be ignorant of, as an amateur, and have been allowed to ignore by a tacit understanding ever since. Both at the hospital and the workhouse I was asked to fill temporarily the place of a lady who never returned to her post, and so the temporary visitor came to be looked upon as the permanent one, whilst still continuing to be indulged in being allowed to visit on all sorts of different days and at all sorts of hours. Although thus favoured, I have tried to take as little advantage of this liberty as possible, and have in the main kept to the same days of the week and the same hours; but still I think it would be well if all visitors to both workhouse and hospital were allowed to pay these surprise visits occasionally, so that they should become better acquainted with the inner workings of these institutions by seeing them in undress, as well as when made spick and span for the visitors, and also because the inmates feel us less of officials when they see we are not strictly bound to days and hours.

To my mind the chief use of visitors to workhouse and hospital is to bring in with them a whiff of outside air, for which reason I discourage as far as possible conversations on ailments and grievances, both at the workhouse and the hospital, and try to make the patients forget, through interesting their minds, the bodily ailments that have brought them into these institutions. Of course, if I find any persons thoroughly preoccupied with the desire to tell me of some specially pressing trouble or ailment, I let them ease their minds of it before trying to arouse their interest in the stirring tale that I do my best to read or relate in the most dramatic manner I can command. Though visiting mainly the women's infirmary wards at the workhouse and the men's medical and surgical wards at the hospital, I have at both institutions made occasional excursions into the wards occupied by members of the opposite sexes, and have found in all cases that the general rule applies that, whereas the women like equally well to hear a tale told or read, you cannot get the men's full attention unless you tell them a story without a book, or talk to them about the events of the day. They agree with the women in liking to discuss with you the points of the story they have heard from your lips, and are always pleased if you show that you consider their opinion of value. If once you can set the men off laughing you will have made them your fast friends; and once they get to like a lady they will vie with each other in their chivalry of manner to her, whilst at the same time expressing their opinions on various subjects with perfect freedom. The women seem more afraid of expressing their opinions, but both sexes are equally grateful for all endeavours on the visitor's part to break down the great dreary blank wall of partition between the classes, and will do their utmost to respond to your advances of friendship if they see that you really want to have them as friends. But they are very keen and discern any attempt at patronising or gaining merit for yourself out of your intercourse with them.

If you set yourself to discover their strong points and draw them out upon these, you will be surprised how much you will learn from them, as you will also be by the literature they show a taste for. I shudder to think how misplaced the insipid tracts must have been that were given by well-meaning ladies to the old paupers at the workhouse, whom I have seen absorbed with intensest interest in the pages of the "Nineteenth Century." I have kept the younger folk of this class on tenter-hooks of eager attention for an hour and a half while I described to them the Empire of the Hittites, and have left them as keen as so many philologists about the decipherment of the script of that ancient race. They love to work up to a subject instead of your talking down to too low a mental level.

My remarks apply to those patients who are not in acute bodily suffering or laid low by severe illness; with these, of course, the merry laugh which it is generally so desirable to elicit, or the sustained interest in outside matters it is in other cases so important to arouse, would be quite out of place. At the workhouse where I have visited, the chaplain was not as often at hand as he might have been, and so I was often called upon to read the prayers for the dying by the bedside of some woman sick unto death; but this would not generally be the experience of a workhouse visitor, and probably never of a hospital one. A few simple words of heartfelt sympathy will always soothe the sick and suffering if you can only make them feel that you suffer in and with their suffering.

I always consider any dogmatic utterances out of place with the dying. Just a few simple words of assurance such as "Trust your Saviour, Who has promised that He will never leave you or forsake you. You must try and clasp Him by the hand while you go through the dark valley; feel that He will be a truer friend to you than ever I have been," will bring more peace at the last than any amount of preaching; and it is just peace these poor things need; for it is, indeed, a hard thing to have to die in public, in the presence of those who are forced in very self-defence to steel themselves against the display of much sympathy with the dying one, seeing that death is so constantly in their midst, that were they to give way to their feelings when each received that last summons, their lives would be passed in perpetual mourning. Not one woman remains alive of all who used to greet me week by week in language so poetical that it would have done honour to our new laureate. I have as it were watched them all die, and thereby have learned how little the physical part of death is to be feared, for in each case what I have called the "dying in public" seemed the worst they had to endure, for not one of them feared death when it came, and very few suffered just at the last.

I feel that my ten years' visiting of the workhouse and hospital has taught me many a lesson of the contentment of the poor and their deep gratitude for the least thing done for them as a friend to a friend; their faces brighten at the sight of the bunch of flowers I always take, or the magazines I lend, quite as much as if I went to them with both hands full of gold, merely because they know I love them with "brotherly love." Not that I claim the slightest merit for so doing, since I cannot help thus loving them, and I tell them the real truth when I say, "You need not thank me, because it is one of the greatest pleasures of my life to visit you."

### Appointments.

The Sisters' Hospital, St. Albans.—Mrs. Elizabeth Haly has been appointed Matron of this hospital. She was trained at Guy's Hospital, and was afterwards on the staff of the Guy's Trained Nurses' Institute. We wish her all success in her new work.

### Nursing: Its Ideals and Realities

### BEFORE AND AFTER.

### Before.

Before I came to be a nurse
I thought no work could be
So gloriously great and grand
As nursing seemed to me.
I thought each Sister was a saint
In sober cap and gown—
A being from some higher sphere
To lower worlds come down.

Before I came to be a nurse
Bright visions filled my mind
Of woman's highest ministry—
Of nurses calm and kind.
I thought the wards were always clean,
The patients good as gold,
And always full of gratitude
And thankfulness untold.

Before I came to be a nurse
I studied what I could,
And mastered all the details that
I thought a novice should.
I went to "First Aid" lectures and
The "Ambulance" exam.,
And did my best the bones and joints,
And all their names, to cram.

Before I came to be a nurse
I thought the uniform
The sweetest dress I'd ever seen;
It took my heart by storm!
I panted for self-sacrifice,
For work that should be real,
And quite forgot the practical
Admiring the ideal!

Before I came to be a nurse
I thought that everyone
Would be so glad to help me on,
And show me what was done.
I thought they all would be my friends,
And take me by the hand,
And welcome me most cordially
To join their happy band.

I thought that nurses never erred,
Nor made the least mistake,
Nor ever minded what they did,
If 'twas for duty's sake.
I never thought of friction there,
Between those happy souls,
Whose bond of union is their work,
And love each act controls.

I thought within those cheerful wards
Was nothing much to do,
But just a dressing here and there
Or poultice to renew.
I thought to sit from morn till night
Beside the couch of pain
And gently bathe the fevered brow
And soothe the troubled brain.

### After.

But afterwards "a change came o'er
The spirit of my dream,"
And very soon I found that things
Are not quite what they seem.
I'd dreamed of gentle ministry
Among the suff'ring poor;
Instead, they made me take a broom
And learn to sweep the floor!

The patients were not nice at all,
And grumbled all day long,
The "gentle" Sisters scolded, too,
When anything went wrong.
I could not do a single thing
Without first asking leave,
Or I was sure a reprimand
Most promptly to receive.

I thought I knew my alphabet,
At least my A.B.C.,
Yet certain cabalistic signs
Were all as Greek to me.
I puzzled over "P.R.N.,"
"O.H.," and "S.O.S,"
But what they meant to signify
I simply could not guess.

My morning's work was well defined,
Though not quite what I thought;
For polishing and trimming lamps
Was not the work I'd sought.
Then, washing babies' pinafores
Was hardly in my line;
And as for pewter inkstands, well,
I could not make them shine.

I went to the dispensary
Some dozen times a day;
I fetched the patients' every meal,
And cleared them all away.
The duster and the scrubbing-brush
Were seldom out of sight,
While story-books and fancy work
Now never saw the light.

And after all I'd tried to learn,
And all the books I'd read,
It was vexatious when I found
I could not make a bed!
No matter what I tried to do
I always did it wrong,
And proved myself the weakest in
The points I thought most strong.

My bandaging was insecure,
My dressings all came off,
My poultices, when made, were such
As made the nurses scoff.
They said I did not mix them well,
The water was not hot.
Oh, dear! the water *I* was in
Was hot, if they were not!

I needed no phrenologist
To point out each defect—
I had so many candid friends
My failings to dissect.
I soon had not a virtue left;
If ever I had one,
It vanished in that hospital,
And left me there with none.

And no one minded in the least
What I might think or say;
The only duty left to me
Was simply to obey,
And not to answer back again,
Or show the least disgust
At being blamed for what, perhaps,
Was after all unjust.

So, bit by bit, it tumbled down,
My castle in the air;
And sadly I surveyed the wreck
Of what was once so fair.
No more a halo of romance
Around my path was shed.
Prosaic details—nothing else—
Filled my bewildered head,

But though I could not help but mourn
My castle in the dust,
It helped me form an estimate
A thousand times more just
Of what a nurse's life should be
From every point of view.
And then I looked with different eyes
On all I had to do.

### Envoi.

And so, in spite of all that's hard,
In spite of tired feet,
There's something in this busy life,
A something that is sweet.
And if I had my choice again,
I still would be a nurse,
And take the cap and apron still
For better or for worse.

### The Present Position of Working Women.

In the magazines and elsewhere many writers have lately drawn attention to "The Position of Women in Industry," and the facts brought out in the reports of the Labour Commission therein commented upon as to the conditions under which the poorer women of England labour to-day are saddening and depressing indeed. Nor does any considerable amelioration of these conditions appear to be within even measurable distance.

"Earnings," it is stated by one writer, "vary more within the different employments than between the different employments. There is an absence of anything approximating to a fixed, or even an average, rate of wages, which is very remarkable; and, with a few exceptions, there is an absence of any great superiority of earnings in one branch of industry over another. An even greater inequality may be noticed in the conditions under which the work is carried on; a factor which, as circumstances at present stand, is of graver importance to the welfare of women than the actual rate of wages." Looking first at the condition of shop-assistants, wages are found to fluctuate between £15 a-year or under, and £100 or more, with board and lodging in each case, the higher rate being far more exceptional than the lower. But the general conditions vary even more than the wages, especially in the length of hours and the degree of consideration shown by employers, this in too many cases being reduced to a minimum indeed. The hours of work at London shops are, as a rule, much longer than those in the provinces, Wales coming out with the most creditable record in this respect and Scotland with the worst. In Scotland the hours of labour extend to 99 and even 102 per week; the usual range averaging from 53 to 79 per week. The sanitary aspect is, however, the worst of all, some employers sinning to a terrible extent in neglecting to provide suitable living and sleeping accommodation. In many cases the effect on a girl's health is disastrous, whilst at most the best that can be said is that it "is not so injurious." Some amount of agitation arose a few years ago with regard to the question of providing shop girls with seats, and a very general impression followed that matters had improved. But this is anything but the true state of the case, and out of a number of shops, very few will be found where the heads of the establishment are sufficiently humane to allow this concession. It is one which must materially affect the health of these girls and it is surely time that something was done to put down so flagrant a piece of tyranny. Why do not some women of position and influence take up the matter and not rest till they have made life a little more bearable for those of their poorer sisters who are unable to stand up for themselves?

The conditions of laundry work are also almost inevitably injurious to health, and the wages are small, the earnings ranging from 6s. to 25s. per week, while the hours of work vary from thirty-six to seventy in the week. Here the public are in fault, for the mistresses of households seldom consider the effect produced on laundry workers by ill-managed domestic arrangements, and the conditions of toilers in this industry might be much ameliorated if more plentiful supplies of linen were the rule in large establishments, thus avoiding a rush of work into two or three days.

The dangers attaching to the trades of match workers and white-lead workers are often very fatal, and here, as in so many cases, it is a terrible and noteworthy fact that "the means of prevention are mainly within the powers of employers"; and that employers do not in too many cases recognise a tithe of their responsibilities. The match-making employers are better in this respect than some, and it is stated that cases of necrosis are found to occur almost entirely amongst makers of non-safety matches. The public would therefore do well to avoid the purchase of such.

Women play a large part in the textile industries of Yorkshire, and it is stated that here "where women predominate largely the men work at women's rates," so that the whole scale of pay is grievously low. The earnings vary from 7s. or 8s. to 20s.

In Lancashire the women earn more as weavers, wages reaching 24s. a week all the year through, but in Lancashire sanitary conditions are worse than in Yorkshire, ventilation and accommodation generally being decidedly bad.

For women serving in places of refreshment long hours are the rule, sometimes being over 100 in the week. It is satisfactory to find that temperance houses never allow the hours to exceed 70 in the week, and that the Aërated Bread Company is endeavouring to bring the hours down to 56, accomplishing this by means of a system of double shifts. As in the case of shop-girls, long standing is here a great evil. Some restaurants give all or a part of the food free, and some pay for washing, a considerable item with the wearing of white collars and cuffs.

The question of the rate of wages for women is a vexed one, and one point is specially borne in upon all who study the matter; this being the undoubted fact that women who work from choice fix both the rate of wages and the standard of work to the very great detriment of the women who work from necessity, a state of things very hard for the latter, and one which it is difficult to see how to remedy. In Birmingham the low rate of wages is accounted for by the employment of married women in factories and workshops, whose husbands are well able to maintain them, and who therefore work for pocket-money or "for an escape from the monotony of home life." This fact holds good not only in these lower branches of women's labour, but in the higher grades as well.

The whole subject is full of difficulty, but one thing seems certain. State legislation can do much, but the efficient organisation of women-workers themselves can do more. Where this is now carried out higher wages are found to follow, and in the final attainment of the needed reforms the efforts of the workers themselves must undoubtedly play a considerable part.

### Novelties for Nurses.

### WATER PILLOWS ON HIRE.

Messrs. Anderson, the well-known waterproof manufacturers, of 37, Queen Victoria Street, E.C., announce a new development in their business. They have now made arrangements for letting out on hire, at reasonable rates, not only water beds, full size, but also "half beds" and water pillows. Messrs. Anderson say they wish to do what they can towards bringing these comforts within the reach of people who could not afford to buy them off hand, and in so doing they will certainly earn the gratitude of many an invalid. All particulars of prices are to be had by writing to the firm. Messrs. Anderson are being very careful with regard to any danger of infection, and require a doctor's certificate with the return bed or pillow clearly stating that it has not been used for any infectious case. Second-hand beds and pillows are also advertised at considerably reduced cost.

### Wants and Workers.

[The attention of correspondents is directed to the fact that "Helps in Sickness and to Health" (Scientific Press, 428, Strand) will enable them promptly to find the most suitable accommodation for difficult or special cases.]

Advice Wanted.—Can anyone advise "An Old Nurse," who writes: "What can a trained nurse do to make a living and provide for an invalid husband? She has been nursing for 20 years, is now 48, very active and industrious, well educated, and of refined habits. Will some kind 'nurse's friend' suggest what she can do? *Not* nursing."

### Everybody's Opinion.

[Correspondence on all subjects is invited, but we cannot in any way be responsible for the opinions expressed by our correspondents. No communications can be entertained if the name and address of the correspondent is not given, or unless one side of the paper only be written on.]

### DISTRICT NURSING COMMITTEES.

"JUSTICE" writes: In justice to what I believe to be the majority of district nursing committees throughout the country, I would like to say that the one of which "Criterion" writes is very exceptional. I have done district nursing for seven different committees (four of which were under the Queen's), so I think I can speak from a fair experience. All these committees had medical men on them, but the practical work was left to the ladies, and there was no mismanagement. Each perhaps had a different way of working, but in no case was I ever asked to go to any house looking for patients or without the doctor's sanction. I think the mistake is often made at the beginning, when the rules of the association admit of anyone sending for the nurse. When the doctors are consulted and put on the committee from the start, and fully understand that the sending of cases rests with them alone, it is much better for everyone. I am sure I carry all the district nurses of the country with me when I say we infinitely prefer cases being sent by doctors. There is nothing more disagreeable and discouraging for a nurse than to go to a patient where she knows the doctor does not want her. As to the question of nourishment, only three out of these seven committees gave any at all, and only in exceptional cases, to parochial patients. Anyone who really knows what parish relief means, knows also that in bad cases it is not enough to get the patient's strength up. I trust I have not encroached too much on valuable space.

[Our correspondent may congratulate herself on her good fortune. There are many committees in existence who still think that an untrained lady or a minister of religion is a fitting person to direct the visits of the district nurse, and to interfere with the local doctor.—ED. *T.H.*]

### NURSING IN CROWN COLONIES.

"SISTER MARGARET" writes: I have just returned from British Guiana, I regret to say, after a failure (from lack of funds) of a very spirited attempt made by Canon Josa to introduce a better system of nursing in the colony. Unfortunately the venture did not meet with the support it merited—partly owing to the general depression among the sugar planters and the scarcity of money, partly to the fact that most of the British population is floating, and principally occupied with the idea of making sufficient to go home. Yet the need is great and appeals to all classes of the community, as many well-educated young men go out annually, principally as overseers, clerks in warehouses, Government officials, &c. Overseers are entitled to medical attendance, and the medical staff (as far as my experience went) were always willing to do their best; but, as we know, without good nursing and constant attention what becomes of the patient? The reply of one of the leading doctors is characteristic. "How do you get your directions carried out?" "They are not; I never expect them to be," and shows the apathetic state of things. The wives and mothers who lose their lives, or suffer permanent injury through carelessness, are very numerous. The mortality among the coloured races is very great, particularly among children. Yet the people are not stupid, but fairly intelligent, and compare favourably with our home poor. They are quick to imitate, and could a proper nursing school be formed pupils would not be wanting, and with care any nurse would be able to stand the climate. Georgetown has a large hospital, 700 beds; Miss Westwood was appointed matron in April, and she has already made great progress towards effecting greater cleanliness among the nurses. At present it is difficult to rely upon them in any way, but no doubt she will in time improve matters, and it is to be hoped the authorities will be able to give her some efficient help. There is rarely any long case of sickness, and very few infectious cases; malarial fever is common, and is often severe. Influenza was epidemic in the early summer months, but rarely so severe as in England. There is no opening for private nurses, unless for anyone with ample means, as living is dear compared to England, and so is rent. Many of the leading ladies would gladly welcome any help, and would do all in their power to aid any feasible scheme.

### MALE NURSES IN AMERICA.

MR. MAGUIRE writes: Referring to Nurse Marie's letter (some few weeks back) *re* male nurses in American hospitals, I can assert that in addition to the "men appearing in every way fitted for the work," male nurses attain to a high degree of proficiency in attendance upon the sick and are second to none as all-round ward nurses. They are well educated, and many of them eventually (as witness several leading American medical men) qualify for the medical profession. The active medical male ward at Roosevelt Hospital in New York, containing fifty beds (mostly full), is entirely managed by three male nurses, who, in addition to ordinary duties, dispense the prescriptions for their own patients. These facts, I think, show that male nurses in American hospitals are useful for other ends than assisting a female nurse to control a "delirious patient" or hold a "man under the influence of drink." Having been an advocate of "male nurses for men" all my life, I write this on behalf of a highly useful class of men, and with pleasant remembrances of "friends across the sea."

### Notes and Queries.

The contents of the Editor's Letter-box have now reached such unwieldy proportions that it has become necessary to establish a hard and fast rule regarding Answers to Correspondents. In future, all questions requiring replies will continue to be answered in this column without any fee. If an answer is required by letter, a fee of half-a-crown must be enclosed with the note containing the enquiry. We are always pleased to help our numerous correspondents to the fullest extent, and we can trust them to sympathise in the overwhelming amount of writing which makes the new rules a necessity. Every communication must be accompanied by the writer's name and address, otherwise it will receive no attention.

### Queries.

(134) *Maternity.*—Will you please tell me of any maternity hospital where I could be trained as a midwife in six weeks, in or near London?—*Rose.*

(135) *Private Home.*—Kindly inform me whether a nurse requires a licence for starting a small home for private patients, and from whom it must be obtained?—*Trained Nurse.*

(136) *Nursing in the Navy.*—Can you give me information about nursing in naval hospitals, and would previous infirmary training be a disqualification to a candidate?—*R. M.*

(137) *Training.*—Where can a well-educated young lady apply for training as a hospital nurse in London or South of England?—*M. S.*

(138) *Missionary Nursing.*—Where shall I apply for information about missionary nurses?—*A. C.*

(139) *Convalescent Home.*—Can you tell me of a convalescent home where a girl in the first stage of phthisis can be placed?—*Sister Edith.*

(140) *Inquirer.*—(1) Where can I obtain a month's good training in a home or hospital in monthly nursing after taking the L.O.S. diploma? (2) What is the best published handbook on the nursing and management of sick children? (3) What is the price of Burdett's "Hospital Annual"?—*H.*

### Answers.

(134) *Maternity* (*Rose*).—No. Three months is the shortest time, and six months is far better.

(135) *Private Home* (*Trained Nurse*).—No licence is required unless you propose to receive certified lunatics. You are probably aware that a private nursing home is a costly undertaking and attended with many risks. Of course you must inform the landlord of the use to which you intend to put the house.

(136) *Nursing in the Navy* (*R. M.*).—Candidates for admission into the naval hospitals must produce certificates of previous training at some recognized institution. Apply by letter to the Director-General, Naval Medical Department, Avenue House, Northumberland Avenue, S.W.

(137) *Training* (*M. S.*).—You do not state your age. Get "How to Become a Nurse" from the Scientific Press, 428, Strand, W.C., which will give you fully the information you desire.

(138) *Missionary Nursing* (*A. C.*).—You do not explain whether you want to know about mission nursing in England or abroad. You might apply to the Nurses' Missionary Association, hon. secretary, Mrs. Leeke, Church House, Weybridge; or to the Zenana Bible and Medical Mission, 2, Adelphi Terrace, London, W.C.

(139) *Convalescent Home* (*Sister Edith*).—You might write for particulars to the All Saints' Convalescent Home at Eastbourne, or the All Saints' Convalescent Home, 8, Pevensey Road, St. Leonards. You will find a complete list of such institutions, with particulars, in "Burdett's Hospital Annual" (Scientific Press, 428, Strand, London W.C.).

(140) *Inquirer* (*H.*).—(1) Write to the Secretary, Midwives' Institute and Trained Nurses' Club, 12, Buckingham Street, Strand, enclosing stamped envelope for reply. (2) "The Mother's Help and Guide to the Domestic Management of Children," by P. Murray Braidwood, M.D., 1s. 11d.. Scientific Press, 428, Strand, W.C. (3) The price of Burdett's "Hospital Annual" is 3s. 9d.

### The Book World for Women and Nurses.

[We invite Correspondence, Criticism, Enquiries, and Notes on Books likely to interest Women and Nurses. Address, Editor, THE HOSPITAL (Nurses' Book World), 428, Strand, W.C.]

THE ART OF LAUNDRY WORK PRACTICALLY DEMONSTRATED FOR USE IN HOMES AND SCHOOLS. By FLORENCE B. JACK. (Edinburgh: T. C. and E. C. Jack; London: Whittaker and Co., White Hart Street, E.C. 1895.)

In the little volume before us Miss Jack has well carried out the purpose set forth in the preface, of supplying full and clearly-expressed instructions in the details of laundry work. A text book it is for laundry classes and a useful guide for those who do their washing at home. It is for the latter purpose that it will be most applicable. The book is arranged in the form of lessons, with clear headings to each section, and very minute and thorough are the directions given upon every branch of the work. Amongst other hints which should be of material service to the home laundress, Miss Jack lays stress upon the importance of putting clothes through some disinfecting process where there has been any illness, "even bad colds." It is, however, a mistake to advocate the use of Sanitas for that purpose in preference to carbolic acid. The diagrams and illustrations will be found a very considerable help in the right understanding of the directions as to ironing and folding, &c., though these are given with admirable clearness of wording. Every housewife who aspires to a well-managed home laundry will find "The Art of Laundry Work" a most useful manual of general reference.

STEAM LAUNDRIES AND PUBLIC HEALTH. (London: The Sanitary Publishing Company, Limited, 2, Fetter Lane, E.C.)

This pamphlet is a considerably enlarged reprint of an article which appeared not long ago in the *Sanitary Record*, and treats of the value as safeguards to the public health of properly inspected and equipped steam laundries. The large steam laundries of to-day are the growth of a surprisingly short time, the general public depending, until a comparatively recent date, almost entirely upon small establishments, often most insanitary in their surroundings, and insufficient in appliances, for the due cleansing and purifying of their household and personal linen. "Clearly to collect large quantities of soiled linen from a number of households and dump it down, often in living rooms, in places badly lighted and badly ventilated; then to mix the linen together and wash it indiscriminately, which was, and still is, the common practice of the cottage laundress, can hardly conduce to health." The author of this article deals fully with the equipment of the various departments of a well-constructed laundry; the precautions that should be taken to keep the workpeople in good health; the disinfection and thorough cleansing of linen and woollen materials; the best machines to effectively carry out the work. The remarks upon the sanitary benefits, which may be looked for as the result of the introduction of electricity as an illuminant, a motive power, and a source of heat, will be heartily endorsed by all who have had practical experience of the enormous advantages it offers. There can be no demur to the concluding statement of the author that "steam laundries, under proper management, offer remarkable guarantees of safety from the health point of view, and therefore they should be welcomed as undoubted aids to public sanitation." . . . They are "real boons of modern city and suburban life." Abstracts from the new Factory and Workshop Act, which has come into force this January, will be found appended to the pamphlet.

### For Reading to the Sick.

### CONSECRATION OF OUR TIME.

**Motto.**

A THINKING man is the worst enemy the Prince of Darkness can have.—*Carlyle.*

**Verses.**

For those thoughts I now atone,
That were of something of my own,
And were not thoughts of Him alone.
—*Lord Houghton.*

Weakest hearts can lift their thoughts to Thee,
It makes us strong to think of Thine eternity.
—*Faber.*

When our thoughts are born,
Though they be good and humble, one should mind
How they are reared, or some will go astray
And shame their mother.
—*J. Ingelow.*

All thoughts that mould the Age, begin
Deep down within the primitive Soul;
And from the many slowly upward win
To One who grasps the Whole.
All thought begins in Feeling—wide
In the great mass its base is hid,
And, narrowing up to Thought, stands glorified—
A moveless pyramid!
Nor is he far astray, who deems
That every hope which rises and grows broad
In the World's heart, by ordered impulse streams
From the great Heart of God.
—*Lowell.*

Fain would my thoughts fly up to Thee,
Thy peace, sweet Lord, to find;
But when I offer, still the world
Lays clogs upon my mind.

Sometimes I climb a little way
And thence look down below;
How nothing, there, do all things seem,
That here make such a show.
—*John Austin.*

My words fly up, my thoughts remain below:
Words without thoughts never to heaven go!
—*Shakespeare.*

**Reading.**

Your manners will depend very much upon the quality of what you frequently think on; for the soul is tinged and coloured with the complexion of thought.—*Marcus Aurelius.*

"There are spaces day by day in almost every life when attention is not demanded for any definite object, when we are or may be free to think of what we will. There are times in which some people are simply listless, and hardly conscious of thinking at all; some build castles in the air; some think of their ambition, or of the scraps of praise that they have heard; some of their anxieties; some of their grievances; some of their dislikes; some, happily, of their hobbies; some, very unhappily, of their health; and some, one must fear, of thoughts that are wholly ruinous and shameful. It is this 'no man's land,' this unclaimed fallow ground, that St. Paul would have rescued from its uselessness or misuse; and he points us to the right and wholesome use for it. "Whatsoever things are true, whatsoever things are honest, whatsoever things are lovely, whatsoever things are of good report; if there be any virtues, and if there be any praise, think on these things." Oh then, for your own sakes, for the sake of your own chief happiness, for the sake of a world that needs your help, for the sake of God, who seeks your love that He may crown your joy, be trying day by day, with watchfulness and prayer, to gain continually more of this high self-mastery in thought; to set the current of your thinking as St. Paul would have it flow; to turn it right away from all impurity, suspicion, sullenness, jealousy, self-deception, or ambition, and to guide it wholly towards those pure and bright and thankful ways where it may pass on surely into the peace of God, into the light of His countenance and the welcome of His love."—"Leisure Thoughts."
—*Dean Francis Paget.*

